# Editorial: Dealing with salinity stress: understanding the mechanism of plant adaptation and resistance

**DOI:** 10.3389/fpls.2023.1278850

**Published:** 2023-09-07

**Authors:** Shufeng Wang, Jianfeng Zhang

**Affiliations:** Research Institute of Subtropical Forestry, Chinese Academy of Forestry, Hangzhou, Zhejiang, China

**Keywords:** salinity stress, plant adaptation, ROS scavenging, selective ion absorption, halophyte

## Introduction

Salt-affected soil, as one of the most widely distributed degraded soil, has attracted global concern for quite a long period, especially in those developing countries with large population and limited arable land (Hussain, 2019; Fukase and Martin, 2020). In order to make better use of these saline-alkali lands, one of the key measures is to select or breed salt-tolerant plants for the purpose of ecological restoration or agricultural production. Therefore, it is necessary to understand the adaptation and resistance mechanisms of plants under saline-alkali stress. The Research Topic on dealing with salinity stress: understanding the mechanism of plant adaptation and resistance brings out multi-angle researches on the recent advances in the mechanisms for salt tolerance and detoxification.

## New advances in the regulation of ROS scavenging and ion homeostasis under salt stress

Effectively scavenging O_2_•^-^ and H_2_O_2_ (ROS) and maintaining ion homeostasis have been considered to be important mechanisms for salt tolerance in plants (Hasanuzzaman et al., 2021; Li et al., 2022). In this Research Topic, we gathered the latest researches on the regulation of ROS scavenging and ion homeostasis under salt stress. For this regard, there are six research articles covering a broad range of plant species, including model plants, crops and herbal medicine species, providing new insight into the physiological and molecular basis of salt tolerance or detoxification in plants.

In the paper “*Arabidopsis AtMSRB5 functions as a salt-stress protector for both Arabidopsis and rice*”, Cai et al. revealed that methionine oxidation and reduction play important roles in plant salt tolerance. They found over expression of *MSRB5*, a type of methionine sulfoxide reductases gene from a salt-stress tolerance 1 (*sst*1) mutant line of *Arabidopsis*, reduced the accumulation of Na^+^ ions in leaves of rice by regulating the stability of H^+^-ATPase 2 (AHA2) and Na^+^/K^+^ homeostasis which led to salt tolerance in both rice and *Arabidopsis*. Similarly, in *Trollius chinensis*, a perennial herbal medicinal plant, Hou et al. revealed that antioxidant defenses play important roles in the response to saline-alkali stress, and they identified a series of new genes encoding key enzymes in the processes of osmoregulation and antioxidation, including chloroperoxidase (CPO), thioredoxin (Trx), and germin-like protein (GLPs), etc.

In addition to the enzymatic antioxidant system, non-enzymatic system also plays an important role in removing hydroxyl radicals and singlet oxygen, which is mainly mediated by low molecular mass antioxidants, such as glutathione, ascorbic acid (AsA) and flavonoids (Gechev et al., 2006). Usually *Apocynum venetum* has been used as a traditional herbal medicine in China to treat angiocardiopathies by regulating blood pressure (Xie et al., 2012) because of the abundance of flavonoids in leaves. Zhang et al. identified and cloned three *A. venetum* flavonoids synthetase genes and conducted genetic transformation of *Arabidopsis thaliana* and confirmed that flavonoids mediate the salt tolerance in *A. Thaliana* by activating the IAA and JA biosynthesis pathways.

Combined salt and drought is usually detrimental to crops. Ali et al. demonstrated that salicylic acid (SA) could mitigate the toxicity of salt in *Brassica napus* (L.) under both drought and salt stresses by relieving membrane lipid peroxidation and minimizing the deterioration of leaf ultra-structures. They also concluded that the combination of drought and salt was synergistic to *Brassica napus* (L.). However, this is not the specific case for halophytes. Notably in the paper “The combination of salt and drought benefits selective ion absorption and nutrient use efficiency of halophyte *Panicum antidotale*”, Hussain et al. revealed that the interaction between high salinity and drought was not detrimental to the survival of *P. antidotale*, in contrast, the combination of high salinity and drought increased the selective ion absorption and improved the nutritional status. This discrepancy indicates that halophytes might possess unique mechanisms different from non-halophytes, especially in extremely high salt conditions.

Chickpea (*Cicer arietinum* L.) is an important legume crop. Although this plant is sensitive to salt stress, considerable variations in salinity tolerance levels were observed among different accessions and cultivars (Vadez et al., 2012; Sweetman et al., 2020). Khan et al. examined the transcriptional difference in two contrasting chickpea (*Cicer arietinum* L.) genotypes (salt-tolerant Genesis836 and salt-sensitive Rupali) and revealed that the different response in two genotypes is attributed to the differential expression of genes involved in ion transport and photosynthesis, especially those transporters for sodium, Na^+^/H^+^ exchanger 1 (*NHX2*—Ca11046) and vacuolar proton-transporting ATPase complex (*VMA21*-like—Ca33135).

## Conclusions and perspectives

In conclusion, the Research Topic deepens the understanding of the mechanisms for salt tolerance and detoxification in various plant species. Certain articles have proved that some salt stress related genes in *Arabidopsis* exhibit the same roles in non-model plants, which might benefit the genetic improvement of crops for planting in salt-affected soils. Further, the linkage between the active ingredients of herbal medicine plants and salt tolerance encourages more thinking about the evolution of salt tolerance or adaptation in herbal medicine plants.

## Author contributions

SW: Conceptualization, Resources, Visualization, Writing – original draft. JZ: Conceptualization, Resources, Validation, Visualization, Writing – review & editing.
